# Short-Term Mild Temperature-Stress-Induced Alterations in the *C. elegans* Phosphoproteome

**DOI:** 10.3390/ijms21176409

**Published:** 2020-09-03

**Authors:** Jichang Huang, Zhen Wu, Xumin Zhang

**Affiliations:** State Key Laboratory of Genetic Engineering, Department of Biochemistry, School of Life Sciences, Fudan University, Shanghai 200438, China; 16210700071@fudan.edu.cn

**Keywords:** phosphoproteomics, *C. elegans*, mild temperature, lifespan, mild stresses

## Abstract

Exposure to mild early-life stresses can slow down aging, and protein phosphorylation might be an essential regulator in this process. However, the mechanisms of phosphorylation-based signaling networks during mild early-life stress remain elusive. Herein, we systematically analyzed the phosphoproteomes of *Caenorhabditis elegans*, which were treated with three mild temperatures (15 °C, 20 °C, and 25 °C) in two different short-term groups (10 min and 60 min). By utilizing an iTRAQ-based quantitative phosphoproteomic approach, 18,187 phosphosites from 3330 phosphoproteins were detected in this study. Volcano plots illustrated that the phosphorylation abundance of 374 proteins and 347 proteins, were significantly changed at 15 °C and 25 °C, respectively. Gene ontology, KEGG pathway and protein-protein interaction network analyses revealed that these phosphoproteins were primarily associated with metabolism, translation, development, and lifespan determination. A motif analysis of kinase substrates suggested that MAPK, CK, and CAMK were most likely involved in the adaption processes. Moreover, 16 and 14 aging-regulated proteins were found to undergo phosphorylation modifications under the mild stresses of 15 °C and 25 °C, respectively, indicating that these proteins might be important for maintaining long-term health. Further lifespan experiments confirmed that the candidate phosphoproteins, e.g., EGL-27 and XNP-1 modulated longevity at 15 °C, 20 °C, and 25 °C, and they showed increased tolerance to thermal and oxidative stresses. In conclusion, our findings offered data that supports understanding of the phosphorylation mechanisms involved in mild early-life stresses in *C. elegans*. Data are available via ProteomeXchange with identifier PXD021081.

## 1. Introduction

Environmental stresses, such as starvation and temperature, have an important effect on animal aging process [1,2,3,4]. Exposure to severe stresses often causes tissue damage and ultimately shortens the lifespan, while mild stresses usually promote defenses against damage and therefore promote longevity [5,6,7]. More importantly, studies in animals have shown that mild early-life stress, such as heat stress, could significantly extend the lifespan of animals [8,9], suggesting that stress in early life could be beneficial for health promotion throughout the animal’s lifespan. However, the exact biological mechanisms behind this phenomenon are largely unknown.

Temperature is a key regulator of the aging process in both heterothermic and homoiothermal animals [10,11], including *Caenorhabditis elegans*, a widely used model animal for aging studies [11,12]. The limiting factors for aging-related research are mainly the long natural lifespan, genetic heterogeneity, and few offspring of most model organisms [13]. *C. elegans* is an excellent aging model thanks to its short lifespan (3–4 weeks) and large numbers of offspring (200–300 animals) [14]. Notably, dozens of aging-related genes are also evolutionarily conserved from *C. elegans* to humans [11]. To date, many aging-related milestone discoveries have been first reported in *C. elegans* [15,16,17]. For example, insulin/IGF-1 signaling (e.g., *daf-2*, *daf-16*, *akt-2*, *daf-18*, and *dao-5*), germ line signaling (e.g., *daf-9*, *daf-12*, *daf-36*, and *glp-1*), and mTOR signaling (e.g., *let-363* and *daf-15*) were uncovered via genetic perturbations in these animals [18]. Since then, plenty of genes have been identified to regulate longevity by influencing responses to a variety of stresses and nutrient alterations [19]. Various genes related to oxidative stress (*clk-1*) [20], mild heat stress (*tax2*/*tax4*) [21], hermetic heat stress (*hsf-1*) [22], and starvation (*pha-4*) [23] are associated with aging.

Nematodes have been cultured under standard laboratory conditions between 15 and 25 °C. The average lifespan of nematodes cultured at 25 °C (high temperature) is considerably shorter than that of nematodes cultured at 15 °C (low temperature) or 20 °C (normal temperature) [12]. However, severe short-term heat stress (36 °C) will not shorten the life of nematodes but will rather prolong life [24]. Notably, in the larval stage and early adult stage, suitable mild heat stress (25 °C), under laboratory conditions, can extend the lifespan of the nematode as well [8,9]. In addition, under mild heat stress (25 °C), heat-sensitive activated *tax-2*/*tax-4* ion channels are distributed in ASJ neurons, and then insulin-like peptides are secreted to inhibit the activity of DAF-16/FOXO, resulting in a shortened lifespan [21]. Moreover, epigenetic regulators (*cbp-1 and swsn-1*) and a stress-activated protein kinase (*pmk-1*) enhance pathogen resistance, promoting longevity following early hermetic heat stress [9]. Overall, these results further indicate that temperature changes occurring in the early stage of life can significantly influence life at later stages, suggesting that proteins’ responses to early temperature changes might be potentially related to the regulation of the life cycle.

Protein phosphorylation, the most widespread type of post-translational modification, is essential for protein function [25,26] and may play an important role in the regulation of late-life activities of nematodes at high temperatures [27,28]. Moreover, phosphorylation is a key regulator that mediates signal conversion in response to external environmental signals for a short time, indicating it might also mediate mild temperature-stress responses in early life. Many studies have achieved the identification and analysis of only a limited number of phosphoproteins, due to the low mounts and technical limitations involved [29,30]. To date, there are no reports of a quantitative atlas of protein phosphorylation driven by different temperatures in early life in nematodes adapting to changing environments. Recently, owing to advancements in proteomics approaches and phosphopeptide enrichment approaches, it has become possible to identify and localize thousands of phosphorylation sites in a single experiment [31].

In this study, the iTRAQ (isobaric tags for relative and absolute quantification)-based proteomic approach [31] was introduced to systematically investigate the phosphoproteomic alterations of L4 nematodes by treating with 15 °C, 20 °C and 25 °C, respectively. A total of 18,187 phosphorylation sites from 3330 proteins were identified. The functional annotations were analyzed to better understand differentially regulated phosphoproteins (DRPs) and major events during early adaption processes. A Motif-X analysis of kinase substrates revealed that MAPK, CK2, and CAMK2 are most likely involved in the adaption processes. We carried out functional validation experiments on four candidates, EGL-27, XNP-1, PAR-1, and SAX-2. It was revealed that for *egl-27-* and *xnp-1-*deficient nematodes, the lifespan was significantly shorter and the stress tolerance was lower than control groups, whereas for *par-1-* and *sax-2-*deficient nematodes, the lifespan did not significantly change but the stress resistance was lower.

## 2. Results

### 2.1. Comparative Phosphoproteomic Analysis of C. elegans under Short-Term Mild Temperature Stress

**Figure 1:**
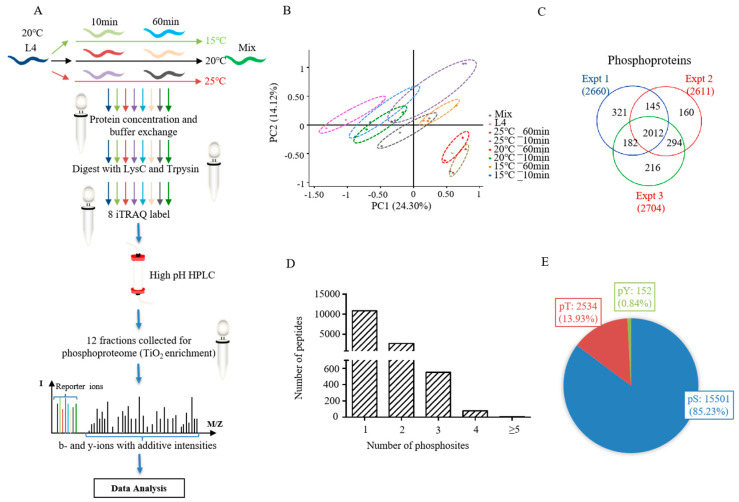
The quantitative phosphoproteome of *Caenorhabditis elegans* exposed to a mild temperature over a short-term period. (**A**) Scheme of the phosphoproteomic strategy. (**B**) Principal component analysis (PCA) of all quantified phosphoproteins. (**C**) The overlap of phosphoproteins in different triplicates of experiments. (**D**) The number distribution of phosphopeptide with different phosphosites. (**E**) The percentage of phosphorylated serine (pSer), threonine (pThr), and tyrosine (pTyr).

The phosphoproteomes of L4 nematodes subjected to different temperatures for different amounts of time were systematically studied by labeling with iTRAQ 8-plex reagents. This included L4 larvals, L4 larvals treated for 10 min or 60 min at 15 °C, 20 °C and 25 °C (six samples), and a mixture of all samples. The labeled peptides were fractionated by HPLC, combined, and then enriched by TiO_2_-affinity chromatography. A total of 12 fractions for each biological replicate were subjected to LC-MS/MS analysis (Figure 1A).

The phosphoproteomic data of all samples were analyzed to evaluate the reliability of experiments using principal component analysis (PCA) (Figure 1B and Supplementary Table S1), which showed similar tendencies of the three biological replicates, indicating high repeatability. From our phosphoproteomic analysis, there were 18,187 phosphosites identified from 3330 phosphoproteins (Supplementary Table S1). Of the three biological replicates, the phosphorylated proteins identified at least twice accounted for 78.4% of all proteins (Figure 1C). In total, 10,822 (76.6%), 2676 (18.9%), and 637 (4.5%) phosphopeptides were identified in one, two, and at least three phosphosites, respectively (Figure 1D). The percentage of phosphosites on phosphorylated serine (pSer), threonine (pThr), and tyrosine (pTyr) were 85.23% (11,501/18,187), 13.93% (2534/18,187), and 0.84% (152/18,187), respectively (Figure 1E).

### 2.2. Identification and Functional Classification of DRPs in C. elegans Responding to Different Temperature

**Figure 2:**
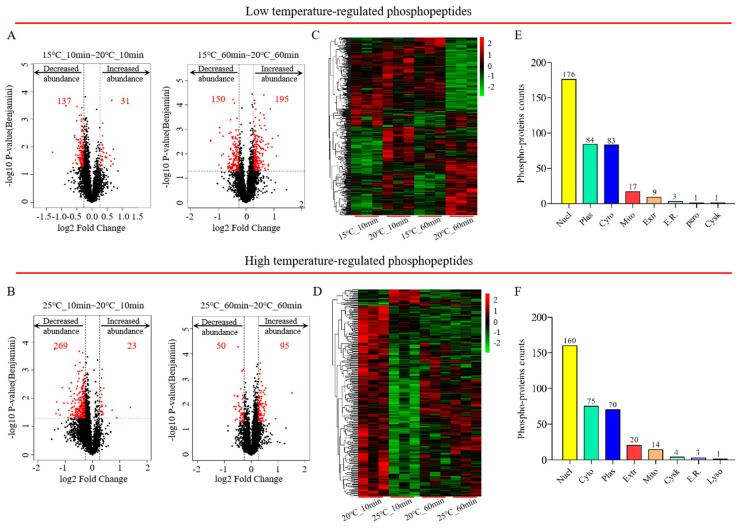
The determination of differentially regulated phosphoproteins (DRPs). Volcano plot analysis of DRPs in *C. elegans* exposed to 15 °C (**A**) or 25 °C (**B**). Heatmap of DRPs in *C. elegans* at 15 °C (**C**) or 25 °C (**D**). Subcellular localization of the phosphoproteins at 15 °C (**E**) or 25 °C (**F**).

To further study the exact biological mechanisms of phosphoproteomes in nematodes exposed to various temperatures over a short period of time, differentially regulated phosphopeptides were graphically shown in volcano plots based on the criteria (fold change ratios ≥ 1.2 or ≤ 0.83 and *p*-value < 0.05). According to the results of the volcano plots (Figure 2A,B and Supplementary Table S2), a total of 500 differentially regulated phosphopeptides were acquired from 374 DRPs in *C. elegans* at 15 °C (217 up-regulated; 283 down-regulated), whilst 428 phosphopeptides were statistically significantly associated with 347 DRPs (115 up-regulated; 313 down-regulated). The significantly altered phosphorylation status of differentially regulated phosphopeptides induced by exposure to various temperatures for 10 min or 60 min was visualized using a heatmap (Figure 2C,D and Supplementary Table S2). The subcellular localization of differential phosphoproteins was intracellular for more than 90% of proteins, of which about 50% of phosphoproteins were located in the nucleus, indicating a rapid response to temperature changes and the regulation of genetic information involved in many biological processes (Figure 2E,F).

**Figure 3:**
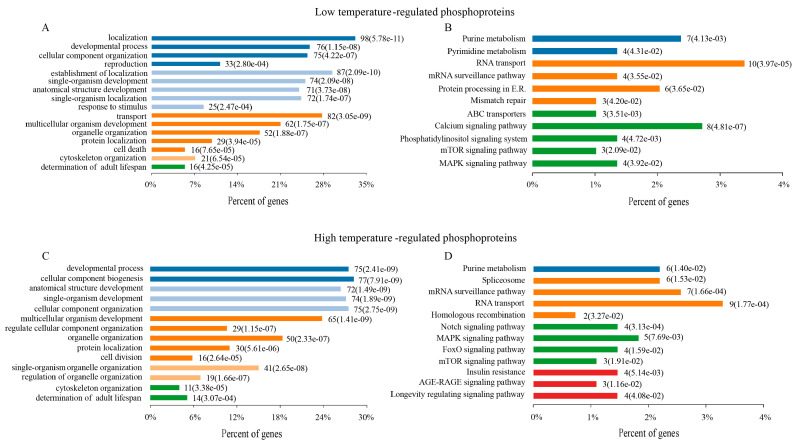
Functional classifications of DRPs. Biological process (BP) enrichment analysis of DRPs at 15 °C (**A**) and 25 °C (**B**). KEGG pathway enrichment analysis of DRPs at 15 °C (**C**) and 25 °C (**D**).

To better understand the physiological functions of DRPs, all of DRPs (10 min and 60 min) were assessed in terms of biological processes (BPs) at 15 °C and 25 °C using gene ontology analysis, respectively. Numerous BPs were found to be significantly altered in *C. elegans* at 15 °C, including development and reproduction, response to stimuli, determination of the adult lifespan, cell death, protein localization, and so on (Figure 3A and Supplementary Table S3). Similar to the BP results, the KEGG pathway enrichment results of these DRPs in *C. elegans* at 15 °C were related to metabolism, RNA transport, proteins proceeding in endoplasmic reticulum, and the longevity pathway (mTOR and MAPK signaling pathway) (Figure 3B and Supplementary Table S3).

In the organisms responding to 25 °C, these DRPs were principally related to many biological processes, including development, determination of adult lifespan, cell division, cellular component and organelle organization (Figure 3C and Supplementary Table S3). The KEGG pathway enrichment results of these DRPs in *C. elegans* mainly indicated the longevity pathway, spliceosome, RNA transport, Notch pathway, mTOR and MAPK signaling pathway (Figure 3D and Supplementary Table S3).

To further explore the functions of DRPs between 10 min and 60 min, a Venn analysis and KEGG pathway enrichment analysis of these DRPs were conducted at 15 °C and 25 °C (Supplementary Figure S1 and Supplementary Table S3). The comparison found that at 15 °C, the functions of 49 DRPs existed in 10 min and 60 min, related to calcium signaling pathway using KEGG pathway enrichment. In terms of different time points, 97 DRPs were found exclusively following 10 min at 15 °C, while 228 DRPs were found exclusively following treatment for 60 min. The KEGG pathway enrichment results revealed that 97 DRPs were related to RNA transport and degradation, calcium signaling pathway, and the longevity pathway (phosphatidylinositol, mTOR, and MAPK signaling pathway), and the 228 DRPs identified at 60 min were mainly involved in purine metabolism, RNA transport, mismatch repair, protein proceeding in endoplasmic reticulum, and the calcium signaling pathway.

Of the DRPs identified after 10 min and 60 min DRPs at 25 °C, 38 were present after both durations, and these related to taurine and hypotaurine metabolism, and non-homologous end joining. 216 DRPs identified at 10 min mainly participated in purine metabolism, RNA transport, protein proceeding in endoplasmic reticulum, and the longevity pathway (mTOR and MAPK signaling pathway). 93 DRPs identified at 60 min mainly related to spliceosome, RNA transport, notch signaling pathway, and AGE-RAGE signaling pathway.

Taken together, the BP and KEGG enrichment analysis results were similar for low- and high-temperature samples, indicating that these physiological functions are driven by extensive phosphorylation alterations following temperature changes.

### 2.3. Protein Protein Interaction Networks of DRPs

**Figure 4:**
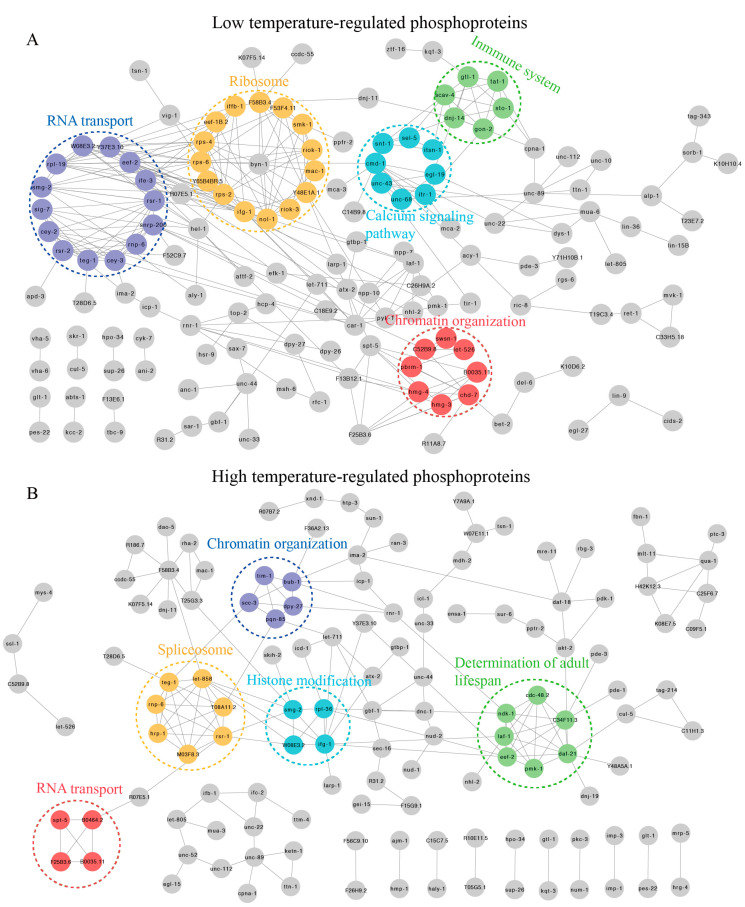
Protein–protein interaction (PPI) networks of DRPs. (**A**) PPI analysis of DRPs in *C. elegans* exposed to 15 °C. (**B**) PPI analysis of DRPs in *C. elegans* exposed to 25 °C.

To provide insight into the possible cellular mechanisms, comprehensive protein–protein interactions (PPI) were used to depict numerous biological aspects of complex regulatory network and signaling pathways [32]. Hence, we used STRING11.0 to construct PPI networks for DRPs at 15 °C. As a result, we discovered that these DRPs might play important roles in translation, RNA transport, chromatin organization, immune system, and calcium signaling pathway (Figure 4A and Supplementary Table S4). In contrast, the highly clustered DRPs at 25 °C were related to spliceosome, the longevity pathway, chromatin organization, and RNA transport (Figure 4B and Supplementary Table S4). These results were similar to those found in the biological processes and KEGG enrichment analyses at 15 °C and 25 °C, further indicating that these processes and pathways may play important roles in early short-term temperature-stress-induced alterations.

### 2.4. Phosphorylation Motifs Analysis in C. elegans Following Short-Term Temperature Stress

Protein phosphorylation is regulated in vivo by protein kinases, which can be predicted using the sequences before and after the phosphosites [29]. To discover the kinases likely responding to low or high temperatures, the motif of protein phosphorylation was further derived from our phosphoproteomic data using the Motif-X algorithm.

**Figure 5:**
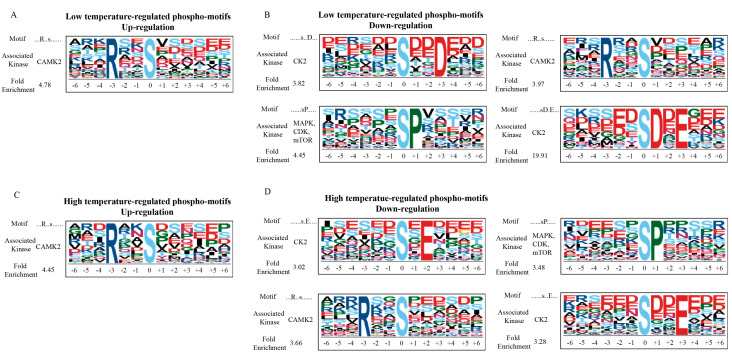
The features of enriched phosphorylation motifs. (**A**) Enriched phosphorylation motif of peptides upregulated at 15 °C. (**B**) Enriched phosphorylation motifs of peptide downregulated at 15 °C. (**C**) Enriched phosphorylation motif of peptides upregulated at 25 °C. (**D**) Enriched phosphorylation motifs of peptide downregulated at 25 °C.

As for the upregulated peptides in *C. elegans* responding to 15 °C, the conserved motif was occupied by a sequence of Arg-ser (the small letters represent the phosphorylated residue). Based on previous studies [33,34], we discovered that calcium/calmodulin kinase II (CaMK2) recognized the “...Rxxs......” sequence (Figure 5A). For the downregulated peptides, the enriched motifs were dominated by the sequences ser-Asp/Glu, Arg-ser, and ser-Pro (Figure 5B). The-e are two motifs recognized by casein kinase II (CK2): “......sxxD...” and “......sD.E...” sequences. These kinases—MAPK, mTOR or CDK—recognize the motifs of the “…...sP.....” sequence. Collectively, our results suggested that CAMK2 was specifically influenced by *C. elegans* in response to a temperature of 15 °C, while CK2 and MAPK, mTOR, or CDK were suppressed, indicating that these protein kinases might be essential regulators at low temperatures.

With regard to the upregulated peptides in *C. elegans* following exposure to a temperature of 25 °C, the enriched motif was occupied by the sequence of Arg-ser (Figure 5C). We found that the “...Rxxs......” sequence is recognized by CAMK2 kinases. As for the downregulated peptides, the conserved motifs were dominated by the sequence of ser-Glu, Arg-ser, and ser-Pro (Figure 5D). The motifs were recognized by CK2 kinase were “......sxE...”, “......sD.E...”. In addition, the motif recognized by CAMK2 kinase and the three kinases MAPK, mTOR, and CDK, including “...Rxxs......” and “…...sP.....”. These results demonstrate that CAMK2 was specifically influenced in the response of *C. elegans* to 25 °C temperatures, while CK2 and MAPK, mTOR and CDK were suppressed, suggesting that these kinases might be involved in the regulation under higher temperatures.

### 2.5. Interaction Analysis of the Kinase-Substrate in C. elegans Induced by Short-Term Temperature Stress

To further explore kinase substrate relationships, a comprehensive kinase substrate phosphorylation network (KSPN) was constructed for the differentially regulated phosphopeptides using iGPS 1.0 software [35].

**Figure 6:**
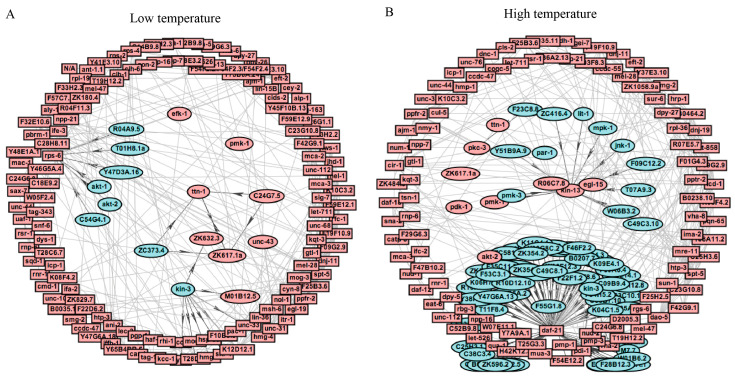
Interaction analysis of kinase substrate relationships of DRPs. (**A**) Kinase substrate interaction analysis of these proteins exposed to 15 °C. (**B**) Kinase substrate interaction analysis of these proteins exposed to 25 °C.

Of the kinases with enriched substrates, 11 kinases were found to have enriched substrate sites and possible high activity in *C. elegans* at 15 °C (Figure 6A and Supplementary Table S5). Similarly, to the results for phosphorylation motifs, the CAMK kinase family, which consists of the kinase TTN-1, ZK617.1a, ZC373.4, and C24G7.5; was identified. The CK2 kinase family member KIN-3 was predicted, and the Ser/Thr protein AGC kinase family, AKT-1, AKT-2, T01H8.1a, C54G4.1, Y47D3A.16, R04A9.5, were identified, indicating that these kinases might be key regulators of low temperature adaptation in *C. elegans*.

With regard to phosphopeptides significantly altered in *C. elegans* at 25 °C, we identified many kinase families, including CAMK, CK1, CK2, and MAPK. The CAMK kinases PAR-1 and F23C8.8, the CK2 kinase KIN-3, and the PLK kinase F55G1.8 were identified (Figure 6B and Supplementary Table S5). Interestingly, the substrates DAF-12 and KIN-13 were found to be regulated by 58 different CK1 kinases and 11 different MAPK kinases, respectively. In-depth studies of this possible kinase-substrate could help to elucidate the effects of short-term temperature-stress on *C. elegans*.

### 2.6. Temperature Responsive Phosphoproteins Associated with the Lifespan of C. elegans

To elucidate the important roles of the temperature responsive phosphoproteins in the regulation of long-term health and lifespan, we focused on the aging-related proteins. As shown in the results of the BP enrichment analysis of differentially phosphorylated proteins responding to low or high temperature in Table 1 and Table 2, lots of aging-related proteins undergo phosphorylation modifications. Interestingly, the aging-related phosphoproteins PMK-1, CNNM-1, NPP-7, and PDE-3 were found at 15 °C and 25 °C, indicating that these proteins might be vital in both stages of the lifespan. A previous study found the serine/threonine kinase PMK-1 [36], which is related to many environmental stresses, in intestine and nerve cells at moderate temperature, while it was shown to translocate into the nucleus at temperature above 32 °C, implicating that the function of PMK-1 is related to temperature changes. Double-knockout CNNM-1 with CNNM-3 resulted in reduced Mg^2+^ levels in other tissues, leading to a shortened lifespan [37].

As for other DRPs, multiple aging-related proteins have been reported to respond to temperature and regulate corresponding life activities. For example, the LMD-3 mutant shortens the lifespan of nematodes at low and high temperatures [38]. The TIR-1 mutant is temperature-sensitive for the AWCON phenotype, with the percentages of AWCON animals being 25%, 58%, and 81% at 15 °C, 20 °C and 25 °C, respectively [39]. NACT-1 modulates broad-spectrum stress resistance against factors such as multiple metals, oxidation, and heat [40]. Moreover, the longevity of worms is regulated by the DAF-12 steroid signaling pathway at high temperatures [41]. More importantly, AKT-2, DAF-18, and DAO-5 are key members in insulin signaling pathway regulating aging process in organisms [42].

These results indicate that early transient temperature changes could affect the longevity signal pathway, which would involve the regulation of physiological activities in response to temperature changes.

### 2.7. Temperature Responsive Phosphoproteins Could Regulate the Lifespan of C. elegans

The physiological functions of early-life DRPs were further explored in relation to the lifespan of *C. elegans*. It was reported previously that a few tissues are associated with aging pathways, e.g., the insulin-like growth factor (e.g., *daf-2*, *daf-16*, *pha-4*, and *skn-1*) pathway in neurons and the germ line signaling pathway [15,16,17,18]. Germ line elimination increases the lifespan of *C. elegans*, and many genes (e.g., *glp-1*, *daf-9*, and *daf-36*) involved in the reproductive system play important roles in longevity [18]. In addition, many conserved genes (e.g., *trpa-1*, *crh-1*, and *tax-2*/*tax-4*) expressed in the nervous system may sense temperature changes and regulate the aging process [8,21,43]. These results indicate that genes expressed in the reproductive and nervous systems might be essential regulators of the lifespan. Therefore, we focused on the candidate DRPs in the above tissues. Egg-laying defective protein 27 (EGL-27) was found to be expressed in the neurons and gonads and was associated with a shortened lifespan under normal temperatures [44]. Sensory axon guidance (SAX-2) was found to be expressed in head neurons and involved in neuron development [45]. Transcriptional regulator ATRX homolog (XNP-1) was found to be expressed in the germ line and it influences vulval development [46]. Serine/threonine-protein kinase (PAR-1) was found to be critical for vulval and neuronal morphogenesis [47,48].

**Figure 7:**
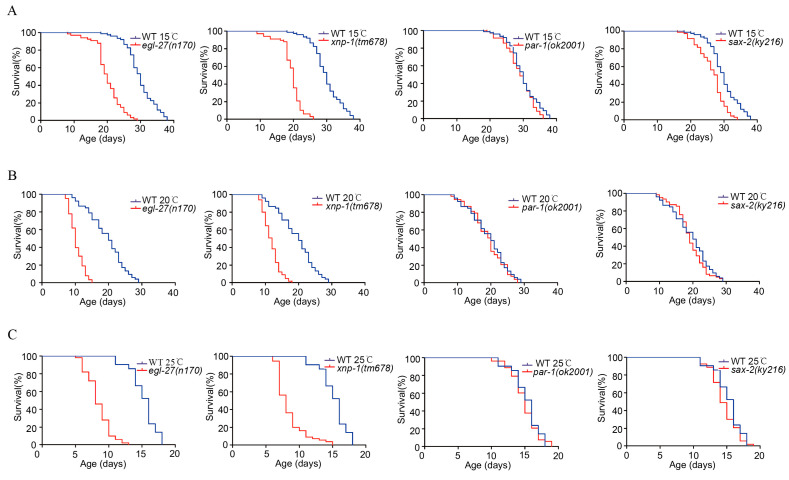
Temperature-responsive phosphoproteins are essential modulators of the worm lifespan. (**A**) The lifespans of *egl-27* and *xnp-1* mutants were significantly reduced at 15 °C (*p* < 0.01, log-rank test). The lifespans of *sax2* and *par-1* mutants were not reduced at 15 °C (*p* > 0.05, log-rank test). (**B**) The lifespans of *egl-27* and *xnp-1* mutants were significantly reduced at 20 °C (*p* < 0.01, log-rank test). The lifespans of *sax2* and *par-1* mutants were not reduced at 20 °C (*p* > 0.05, log-rank test). (**C**) The lifespans of *egl-27* and *xnp-1* mutants were significantly reduced at 25 °C (*p* < 0.01, log-rank test). The *sax2* and *par-1* mutants were not significantly reduced at 25 °C (*p* > 0.05, log-rank test).

Based on these conditions, the candidate phosphoproteins (EGL-27, XNP-1, PAR-1, and SAX-2) were further chosen to confirm their effects on the longevity of *C. elegans*. The lifespans of EGL-27, XNP-1, PAR-1, and SAX-2 mutants were detected at 15 °C, 20°C and 25 °C (Figure 7). As shown in Figure 7, EGL-27 and XNP-1 mutants significantly shorten the lifespan compared with the wild-type nematodes (*p* < 0.01), while PAR-1 and SAX-2 are not significant compared with wild-type (*p* > 0.05), indicating the critical functions of EGL-27 and XNP-1 in regulating the lifespans of these worms.

**Figure 8:**
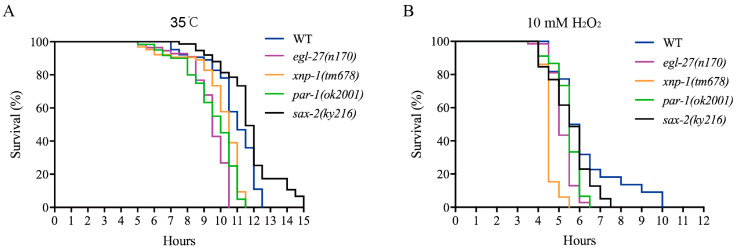
Temperature-responsive phosphoproteins are important regulators of the lifespan of *C. elegans*. (**A**) One-day-old *egl-27*, *xnp-1*, and *par-1* mutants were less resistant to heat stress (*p* < 0.01, log-rank test). One-day-old *sax-2* mutants showed thermal resistance (*p* < 0.01, log-rank test). (**B**) One-day-old *egl-27*, *xnp-1*, and *par-1* mutants were shorter than wild-type strains under hydrogen peroxide treatment (*p* < 0.01, log-rank test). One-day-old *sax-2* mutants were shorter than wild-type strains under hydrogen peroxide treatment (*p* < 0.05, log-rank test).

Lifespan extension has a certain correlation with resistance to different stresses, e.g.; thermal and oxidative stresses [49,50]. Survival curve analysis is typically used to evaluate the impacts of various stresses on organisms. Under thermal stress (35 °C) or hydrogen peroxide induced oxidative stress, the survival curves of EGL-27, XNP-1, and PAR-1 mutants were found to be shorter than those of wild-type strains (*p* < 0.01) but not that of SAX-2 mutants (Figure 8A,B). Taken together, EGL-27, XNP-1, and PAR-1 confer resistance to various stresses, improving survival rates.

## 3. Discussion

Many studies have studied the effects of mild stresses on extending the lifespan [8,9]. However, very little work has investigated how short-term mild stresses could have long-term beneficial effects. Herein, we conducted a systematic phosphoproteome analysis to identify and quantify the transient phosphoproteins of nematodes in response to different short-term temperature treatments. Functional experiments further confirmed that the responses of the early proteins EGL-27, XNP-1, and GTBP-1 to temperature, could regulate the lifespan of *C. elegans* at 15 °C, 20 °C and 25 °C.

As mentioned above, a total of 18,187 phosphorylation sites from 3330 proteins were recognized and the phosphosite distribution of pSer, pThr, and pTyr was consistent with that of other organisms [29], suggesting that protein phosphorylation is a highly conserved modification. A volcano plot revealed 374 DRPs in *C. elegans* at 15 °C, whilst there were 347 DRPs at 25 °C. More than 90% differentially expressed phosphoproteins were located in cells, of which about 50% were in the nucleus. Using BP and KEGG analysis, these phosphoproteins were found to be mainly involved in spliceosome and RNA transport. This suggests that the rapid response to external temperature changes involves dynamically changing phosphorylation modifications that regulate genetic information in many biological processes. In addition, other processes, including development, reproduction, and determination of the adult lifespan, were found to be enriched at 15 °C and 25 °C. Our results are compatible with the reported temperature regulation of physiological activities in organisms [41,51,52].

Notably, differentially regulated proteins responding to low temperature or high temperatures in the early nematode life stages are involved in the regulation of several conserved longevity pathways. PMK-1 is one kind of MAPK that affects the cascade reaction of MAPKs and could regulate meiosis and mitosis in cells during the aging process [53]. Moreover, the essential regulators of the insulin-signaling pathway, AKT-2, DAF-18, and DAO-5, can regulate the aging process [11]. In addition, DAF-12, an important component of reproductive system signaling, may regulate the process of high-temperature aging [41]. RPL-19 and RPS-6, which are related to the ribosome protein synthesis machine, can regulate proteomic synthesis, thus affecting the aging process [54]. Furthermore, it has also been reported that proteins that respond to temperature changes, such as LMD-3, can regulate the lifespan of *C. elegans* [38]. These results offer phosphoproteome-wide datasets that can deepen our understanding of early thermal processes.

The motif-substrate and kinase-substrate phosphorylation network results showed that CK, CAMK, AGC, MAPK, mTOR, and CDK are involved in early-life of nematodes’ responses to short-term temperature change. The kinase CK2 can regulate various biological processes, including gene expression and DNA repair, and can retard DNA damage and ensure genome integrity [55], thus delaying aging [54]. In addition, the reduction of protein synthesis improves protein balance, and thus extends longevity [54]. Several lines of evidence indicated that the early thermal-stress kinase CK2 might be an important regulator of long-term health benefits in nematodes. Moreover, MAPK, mTOR, and CDK are associated with multiple biological processes, e.g., immunity and stem-cell regeneration, accompanying the aging process [56,57]. Failure to defend against harmful factors may be due to a decline in immunity and could accelerate aging [54]. Furthermore, CAMK2 can impact cell growth and proliferation by regulating the calmodulin-induced reaction cascade [58]. It has been reported that the growth and proliferation of stem cells is significantly reduced during the aging process [59]. More importantly, these kinases might be essential regulators in the later life [28]. In brief, these kinases may play essential roles in the regulation of phosphorylation events throughout the lifespan.

The phosphoprotein EGL-27 (egg-laying defective protein 27) is a transcription factor that promoted stress survival and delayed aging at 20 °C [44]. We found that EGL-27 also modulated the lifespan of nematodes at both 15 °C and 25 °C. In addition, the phosphoprotein XNP-1 (transcriptional regulator ATRX homolog) mutant is temperature-sensitive, with 38% embryonic lethality at 25 °C [46]. The XNP-1 mutation significantly shortened the lifespans in nematodes at 15 °C, 20 °C and 25 °C and was associated with resistance to multiple stresses. These results add our understanding of the early temperature-sensitive phosphoproteins’ functions in later life.

In conclusion, our phosphoproteomic study systematically explored the effects of phosphorylation evens in response to early-life temperature changes. The functions of EGL-27 and XNP-1 modulated the lifespan upon exposure to different temperature. Nevertheless, further studies are needed to explore the mechanisms associated with these phosphoproteins. Our results offer phosphoproteome-wide data that will aid in the elucidation of short-term stress effects on long-term health benefits.

## 4. Materials and Methods

### 4.1. Worm Culture

The worms were cultured in accordance with the method presented in a previous study [60]. wild-type N2, MT170 *egl-27 (n170)* II., IG256 *xnp-1 (tm678)* I., RB1625 *par-1 (ok2001)* V., and CX3385 *sax-2 (ky216)* III. strains were purchased from the Caenorhabditis Genetics Center (CGC) (University of Minnesota, Minneapolis, MN, USA). Strains were placed onto agar plates with *E. coli* OP50 as food.

### 4.2. Phosphoproteomic Sample Preparation

To achieve age-synchronized L1 larval cells, eggs were originally obtained from gravid worms in accordance with a previous study [61]. The L1 larval cells were cultivated on NGM plates at 20 °C. The L4 nematodes were derived from age-synchronized L1 larval cells. In the late L4 stage, nematodes were separated into three groups and treated at 15 °C, 20 °C and 25 °C, respectively. The late-stage L4 nematodes growing at 20 °C were treated at 15 °C, 20 °C and 25 °C for 10 min and 60 min, and then immediately frozen to liquid nitrogen, followed by storage at −80 °C.

### 4.3. Protein Sample Preparation, Enzymatic Digestion, and iTRAQ Labeling

Briefly, the samples were resuspended in lysis buffer (4 M Gnd·HCl, 100 mM TEAB, 1% protease and phosphatase inhibitor cocktail). The samples were sonicated for 6 min on ice in 5 s bursts with pauses of 2 s. The samples were centrifuged for 20 min at 20,000× *g* and 4 °C. The supernatant was collected, and the protein concentration was measured with the Bradford assay. The samples were reduced, alkylated, and digested by Lys-C (MS grade, Wako Chemicals, Osaka, Japan) and trypsin (MS grade, Promega, Madison, WI, USA), as previously described [62]. Eight-plex iTRAQ reagents (AB Sciex, Foster City, CA, USA) were used to label digestive peptides based on the manufacturer’s instructions. The peptides (L4, 15 °C_10 min, 20 °C _10 min, 25 °C_10 min, 15 °C_60 min, 20 °C _60 min, 5 °C_60 min, and the mixture of the seven samples) were marked by 113, 114, 115, 116, 117, 118, 119, and 121-reagents, respectively.

### 4.4. Peptide Fractionation with HPLC and TiO_2_ Affinity Chromatography Enrichment

The labeled peptides were pooled with the same amount of each and then fractionated using a Dionex UltiMate-3000 HPLC system (Thermo Fisher Scientific, MA, USA). A high-pH reverse-phase separation was employed as the first dimensional separation. The peptides were passed through a C18 analytical column (2.6 µm, 100 mm × 3 mm (Phenomenex, Torrance, CA, USA)) through Solvent A (10 mM NH_4_Cl, pH 10.0) and Solvent B (10 mM NH_4_Cl, 80% ACN). Three hundred microgram samples of the generated peptides were loaded onto the column using Mobile Phase A, and the flow rate was set as follows: 0.1 mL/min for 10 min, 0.1–0.2 mL/min for 1 min and 0.2 mL/min for 34 min. The separation gradient was set using Mobile Phase B as follows: 0% for 11 min, 0–40% in 25 min, 40–100% in 2 min, 100% for 2 min, 100–0% in 2 min, and 0% for 2 min. Fractions were collected from the 6th min with 1 min intervals over the following 40 min. Forty fractions were collected and mixed into 12 fractions. The mixed fractions were lyophilized and enriched followed by TiO_2_-affinity chromatography in accordance with previous study [63].

### 4.5. Mass Spectrometry Analysis

The LC-MS/MS assay was conducted in a nanoflow EASY-nLC 1000 system (Thermo Fisher Scientific, Odense, Denmark). The peptides were separated using an Acclaim PepMap100 C18 Column (5 μm, 100 μm i.d. × 2 cm, 100 Å). The separated peptides were examined using an Acclaim PepMap RSLC C18 column (2 μm, 100 Å, 75 μm i.d. × 25 cm; Thermo Fisher Scientific, Sunnyvale, CA, USA). The LC was coupled with a LTQ Orbitrap Elite mass spectrometer (Thermo Fisher Scientific, Bremen, Germany).

The mobile phases contained Solvent A (0.1% formic acid) and Solvent B (0.1% formic acid in ACN). The separated peptides were set using Solvent B as follows: 2–5% B in 11 min, 5–28% in 98 min, 28–35% in 5 min, 35–39% in 2 min, 98% for 13 min at a flow rate of 200 nL/min. The data collection method was fixed to acquire a 1 MS scan with a resolution of 60,000 (*m/z* 400–1600, automatic gain control (AGC) target of 1e6, and a maximum ion injection time (IIT) of 100 ms), and 15 MS/MS scans of the strongest ion were detected by HCD (normalized collision energy (NCE) of 35; isolation width of 2 *m/z*; resolution of 15,000; AGC target of 5e4 and maximum IIT of 100 ms). Repeated peptides were dynamically excluded of 60 s. The precursor mass tolerance was set at 10 ppm.

### 4.6. Database Searching and Analysis

The uniprot worms database (20161228, 17,392 sequences) was searched for raw data using Proteome Discover (Version 1.4, Thermo Fisher Scientific, Bremen, Germany) followed by scoring with the Mascot server (Version 2.3, Matrix Science, London, UK). The searched parameters were set as follows: 10 ppm for the precursor ion mass deviation and 0.05 Da for the allowed fragment mass deviation; up to two miscleavage sites; cysteine carbamidomethylation, iTRAQ 8-plex on lysine and the N-terminal set as fixed modifications; methionine oxidation; and STY (serine, threonine and tyrosine) phosphorylation as variable modifications. The Target Decoy PSM verification program incorporated in the Proteome Discoverer was uitilized to verify the search results using only matches with FDR ≤ 0.01. Functional classifications of phosphoproteins were analyzed using the David database, as previously described [64]. Subcellular locations were predicted through Wolf PSORT [65]. Protein–protein interactions were detected by STRING [32]. The motifs of protein phosphorylation were derived from our phosphoproteomic data using the Motif-X algorithm [66]. The kinase-substrate phosphorylation network (KSPN) was constructed by submitting differentially expressed phosphopeptides by using iGPS 1.0 software (the CUCKOO workgroup, DICP, Dalian, China) [35].

### 4.7. Lifespan and Stress Assays

The lifespans of nematodes were monitored at 15 °C, 20 °C and 25 °C. Herein, one-day-old worms were considered to be at the first day of adulthood. Nematodes were recorded as dead in the absence of pharyngeal pumping and no response to gentle contact with a platinum wire. Worms that died of causes other than aging, such as sticking to the plate walls, bursting in the vulval region, or forming worm bags, were excluded from our analysis. With regard to each lifespan assay, 50–80 nematodes were cultured on NGM plates. All assays were carried out in triplicate with 50–80 worms in each replicate. To monitor the effects of temperature on longevity, L4-stage strains were cultured at 20 °C, and shifted to 15 °C, 20 °C and 25 °C.

For stress assays, One-day-old strains were placed onto agar plates at 35 °C and then the lifespans were recorded. One-day-old strains were transferred into S-basal buffer containing 10 mM of hydrogen peroxide and then the lifespans were recorded. All assays were carried out in triplicate using 40–80 worms in each replicate. The log-rank (Kaplan–Meier) test was used to calculate *p*-values. The survival curves were drafted using GraphPad Prism 8 (GraphPad Software Inc., San Diego, CA, USA).

## Figures and Tables

**Table 1:** The aging-related phosphoproteins in *C. elegans* at low temperatures.

Accession	Gene Names	Sequence	Log2FC	Protein Names
O17003	*natc-1*	EYEGAEENSDEKSpEESpTSDPTPSSEAK	−0.614	N-alpha-AcetylTransferase C complex subunit
O02639	*rpl-19*	VWLDPNEVSpEISGANSR	−0.456	60S ribosomal protein L19
Q17446	*pmk-1*	QTDSEMTpGYpVATR	−0.416	Stress-activated protein kinase pmk-1
O61955	*ife-3*	GPAATTSPSNPGTEATGTSPATpPTpP	−0.369	Eukaryotic translation initiation factor 4E-3
P91457	*npp-7*	TASTASTTTINSpSNSR	−0.315	Nuclear Pore complex Protein
Q18212	*hel-1*	LANDCPHIVVGTpPGR	−0.28	Spliceosome RNA helicase DDX39B homolog
Q8I0P7	*pde-3*	KESpGTHVDTVVTTIDGQR	−0.267	Probable 3’,5’-cyclic phosphodiesterase
O45865	*ant-1.1*	LLLQVQDASpK	0.273	Adenine Nucleotide Translocator
H2L003	*lmd-3*	SpIQGTVTSGAEK	0.296	LysM Domain (Peptidoglycan binding) protein
E5QCE7	*dnj-14*	EESpPAADHSHDPK	0.31	DNaJ domain (Prokaryotic heat shock protein)
Q9NEN6	*rps-6*	SSpASpHHSpESEVK	0.327	40S ribosomal protein S6
A3QM97	*cnnm-1*	SMSpIVGTSELSAR	0.396	Metal transporter cnnm-1
Q21219	*pept-1*	GYSESRSpESpVSpSK	0.463	Peptide transporter family 1 (Di-/tri-peptide transporter CPTB)
G5EFL5	*alp-1*	AAYHPQVNTpARPVSpVSpPAPSpAGSK	0.478	ALP/Enigma encoding
H2KZZ2	*hlh-30*	QVVSSSpAPTSSIDIEK	0.654	Helix Loop Helix
Q86DA5	*tir-1*	FLIQQDSpVVNPSTK	0.671	Sterile alpha and TIR motif-containing protein

**Table 2:** The aging-related phosphoproteins in *C. elegans* at high temperatures.

Accession	Gene Names	Sequence	Log2FC	Protein Names
G5EFF5	*daf-12*	LEESSGACGSpPDDGLLDSSEESR	−0.606	Nuclear hormone receptor family member
Q18688	*daf-21*	IEGAEEDASpRMEEVD	−0.477	Heat shock protein 90
O45962	*rle-1*	DNSHNSpPQTpPPK	−0.394	Regulation of longevity by E3 ubiquitin-protein ligase
Q9XVS4	*dao-5*	ADLSSDFSpDDEAPAK	−0.393	Dauer or Aging adult Overexpression
G5EE01	*daf-18*	VTpPPPDVPSTSTR	−0.343	Phosphatidylinositol 3,4,5-trisphosphate 3-phosphatas
Q8I0P7	*pde-3*	RDSpPLDSDLSQ	−0.333	Probable 3’,5’-cyclic phosphodiesterase
Q9XTG7	*akt-2*	EFTSMPVQLTpPPR	−0.329	Serine/threonine-protein kinase
P91457	*npp-7*	TASTASTTTINSpSNSR	−0.329	Nuclear Pore complex Protein
Q8MQ70	*hpk-1*	NSpGQSTDLNSR	−0.322	Homeodomain-interacting protein kinase 1
O76360	*egl-4*	KPSpDQPNGNQVQVGTR	−0.271	cGMP-dependent protein kinase
A3QM97	*cnnm-1*	SMSpIVGTSELSAR	0.34	Metal transporter
O18696	*pde-1*	SpYDNAPALESLEK	0.403	Probable 3’,5’-cyclic phosphodiesterase
Q10663	*icl-1*	AGSpVVNRIPEAADLLEK	0.414	Bifunctional glyoxylate cycle protein
Q17446	*pmk-1*	QTDSEMTpGYpVATR	0.537	Stress-activated protein kinase

## Supplementary Materials

The Supplementary Materials are available online at https://www.mdpi.com/1422-0067/21/17/6409/s1.

## Abbreviations

BP	biologic process
CaMK II	calcium/calmodulin kinase II
CDK	cyclin-dependent kinase
CK	casein kinase
DRP	differentially regulated phosphoprotein
FDR	false discovery rate
HPLC	high performance liquid chromatography
iTRAQ	isobaric tag for relative and absolute quantitation
KEGG	Kyoto encyclopedia of genes and genomes
LC-MS/MS	liquid chromatography-tandem mass spectrometry
MAPK	mitogen-activated protein kinase
mTOR	mammalian target of rapamycin
PCA	principal component analysis
PPI	protein-protein interactions
