# Supplementary figures and images for: Short-Term Mild Temperature-Stress-Induced Alterations in the C. elegans Phosphoproteome
Source: Int J Mol Sci. 2020 Sep 3;21(17):6409. doi: 10.3390/ijms21176409 (PMC7504583; doi:10.3390/ijms21176409)

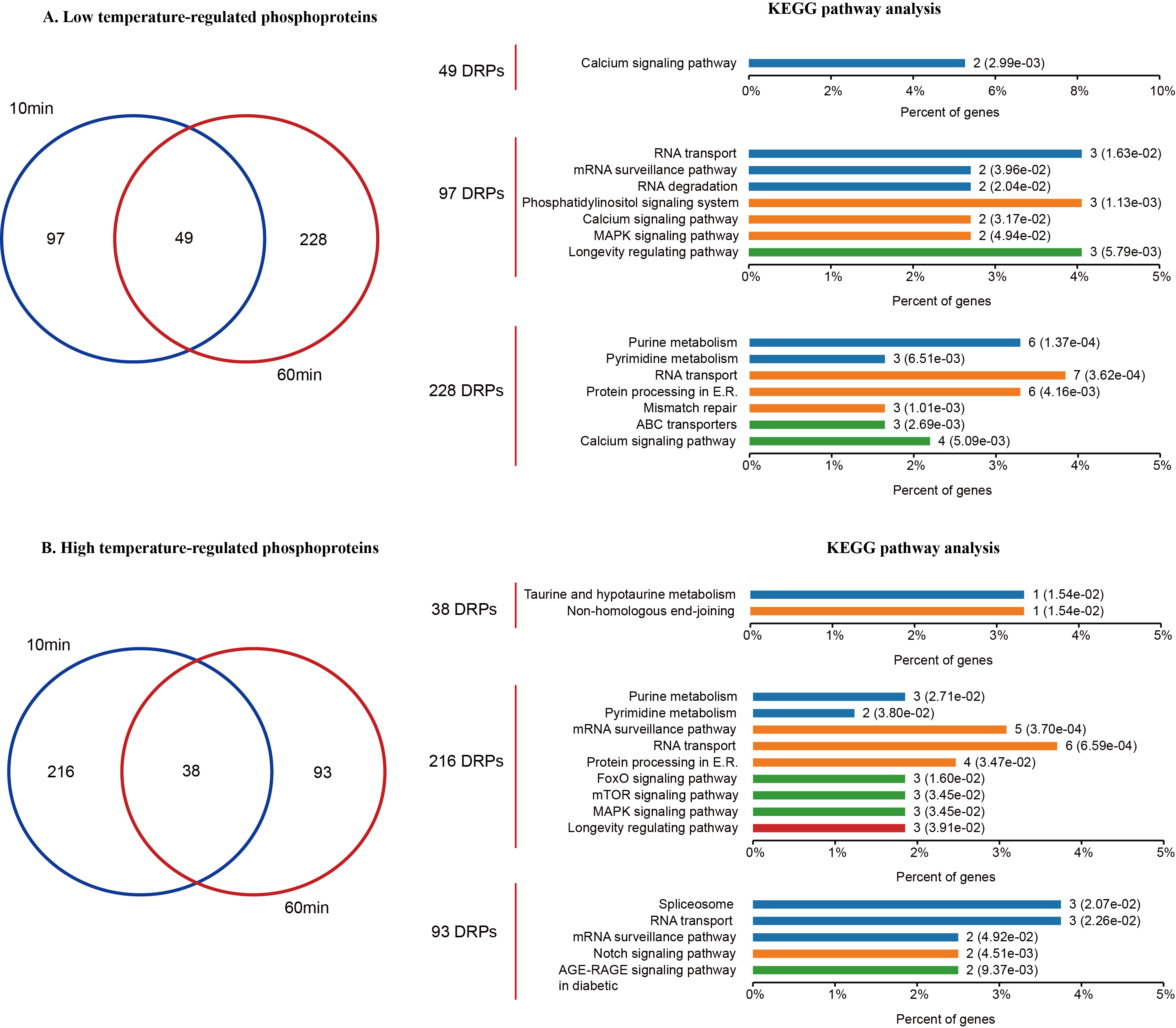

Supplement: Supplementary file 1 [file ijms-21-06409-s001.zip › Supplementary/Supplementary Figure S1.png]
